# Acute-on-Chronic Carbamazepine Overdose: A Toxicology Simulation Case for Fourth-Year Medical Students

**DOI:** 10.15766/mep_2374-8265.11640

**Published:** 2026-10-14

**Authors:** Lauren Leung, Krithika Senthil, John Kothmann, Johnny Bui, Derick Nguyen, Caelan Nzamba, Michelle Troendle

**Affiliations:** 1 Medical Student, Virginia Commonwealth University School of Medicine; 2 Medical Student, Virginia Commonwealth University School of Medicine; 3 Medical Student, Virginia Commonwealth University School of Medicine; 4 Medical Student, Virginia Commonwealth University School of Medicine; 5 Medical Student, Virginia Commonwealth University School of Medicine; 6 Medical Student, Virginia Commonwealth University School of Medicine; 7 Associate Professor, Department of Emergency Medicine, and Attending Physician, Division of Clinical Toxicology, Virginia Commonwealth University Health

**Keywords:** Carbamazepine, Overdose, Simulation, Multidose Activated Charcoal, Emergency Medicine, Internal Medicine, Medical Toxicology, Neurology

## Abstract

**Introduction:**

Carbamazepine is an antiepileptic medication used to treat seizures, trigeminal neuralgia, and bipolar disorder. Although carbamazepine overdose occurs frequently, it receives limited attention in undergraduate medical curricula. Multimodal, experiential learning provides a safe, structured learning environment. We developed a simulation of carbamazepine overdose to address this educational gap.

**Methods:**

Fourth-year medical students enrolled in a toxicology course completed a pretest and a Likert-scale questionnaire assessing confidence in managing carbamazepine overdose. Students received a 30-minute lecture and participated in a 15-minute simulation involving a 26-year-old patient with an intentional carbamazepine overdose followed by a 15-minute debrief. Critical actions included obtaining a focused history, neurological assessment, and carbamazepine level; administering multidose activated charcoal; and requesting toxicology and psychiatric consultation. Required equipment included a high-fidelity manikin, IV supplies, and standard pharmaceuticals. We evaluated students with a critical actions checklist and posttest. Students then completed additional Likert-scale questionnaires assessing workshop reactions, confidence, and intent to change practice.

**Results:**

Forty-nine students participated across 10 sessions. Mean pretest and posttest scores were 67% (*SD* = 19) and 98% (*SD* = 5), respectively, with significant improvement (Wilcoxon W = 0.0, *P* < .001). All groups completed the critical actions checklist. Median confidence scores for identifying clinical manifestations, ordering and interpreting diagnostic studies, and initiating treatment increased from 3’s and 4’s to 5 across all domains (Mann-Whitney U *P* < .001). Students rated the workshop favorably and most reported intent to change practice.

**Discussion:**

Experiential learning is an effective pedagogical technique to teach carbamazepine overdose.

## Educational Objectives

By the end of this activity, learners will be able to:

1. Identify the pharmacokinetics and toxicokinetics of carbamazepine relevant to overdose management.
2. Perform the clinical tasks required to evaluate a simulated patient with carbamazepine overdose, including obtaining a focused toxicologic history, performing a targeted physical examination, and requesting a neurological examination.
3. Interpret clinical findings and laboratory results to identify manifestations of carbamazepine toxicity.
4. Implement evidence-based management steps for carbamazepine overdose using a critical actions checklist, including management and consultation with appropriate services.

## Introduction

Carbamazepine is a widely prescribed antiepileptic medication used to treat partial seizures, generalized tonic-clonic seizures, and mixed seizure patterns, as well as trigeminal neuralgia and bipolar disorder.^1^ It has a narrow therapeutic index, which increases the risk of toxicity, with even modest supratherapeutic doses capable of causing serious adverse effects.^2^ Neurological manifestations predominate in overdose scenarios, presenting as movement disorders, altered mental status, seizures, and coma.^3^ In more severe cases, cardiovascular and respiratory complications such as tachycardia, hypotension, myocardial depression, and respiratory compromise may occur.^3^ Because carbamazepine undergoes extensive hepatic metabolism—primarily through cytochrome P450 3A4 (CYP3A4)—and strongly induces multiple drug-metabolizing enzymes, it is associated with a wide range of drug-drug interactions.^2,4^ Genetic variations affecting metabolic pathways, as well as interacting medications that alter carbamazepine metabolism, can further increase the risk of toxicity.^2^

According to the 2023 Annual Report of the National Poison Data System from America's Poison Centers, 2,011 toxic exposures to carbamazepine were reported.^5^ Therefore, carbamazepine overdose is relatively common, but it is often overshadowed by other more common overdoses in medical curricula, resulting in a knowledge gap. Traditional enhanced elimination methods, such as dialysis, are less effective due to carbamazepine's high protein binding (70%-80%) and large volume of distribution, and should be reserved as a supplemental method of enhanced elimination for severe, life-threatening overdoses.^6^ However, carbamazepine's enterohepatic recirculation makes it susceptible to enhanced elimination via multidose activated charcoal.^7^ Given the frequency of carbamazepine overdose, its increased risk of toxicity due to drug-drug interactions and genetic variations affecting metabolism, its potential for severe clinical consequences, and the need to understand less commonly utilized methods of enhanced elimination, medical students must be well prepared to manage these cases effectively.

Experiential learning in simulation labs provides students with a structured environment to develop essential skills, including interprofessional communication, history taking, physical examination, procedural techniques, clinical reasoning, troubleshooting, and escalation of care in a safe learning environment.^8^ Previous research has explored this teaching methodology in the context of muscle rigidity secondary to tetanus.^9^ In this study, students engaged in experiential learning through a simulated tetanus case after listening to a recorded lecture on its pathophysiology.^9^ The results demonstrated a statistically significant improvement in test scores assessing tetanus pathophysiology between pre- and postexperiential learning.^9^ Additionally, high-fidelity simulation has been shown to improve clinical training outcomes across multiple health care disciplines and to improve nontechnical skills of communication and teamwork.^10^ To address the knowledge gap surrounding carbamazepine toxicity, we adapted this pedagogical approach to a new clinical scenario by developing a simulation on carbamazepine overdose for medical students. The simulation functioned as both an instructional and assessment modality. To our knowledge, there are no prior examples of simulation cases of carbamazepine overdose within *MedEdPORTAL* or other journals.

## Methods

### Activity Design and Development

We developed this simulation for fourth-year medical students rotating through their toxicology elective, as carbamazepine overdose is common in clinical practice but receives limited coverage in undergraduate medical education. We implemented a lecture and simulation and measured learner knowledge gain using pre/posttest assessments. We used the Understanding by Design framework model of curriculum development^11^ to guide the project and ensure alignment with assessment methods and learning objectives. This activity was designed by Dr. Michelle Troendle, director of the clinical toxicology elective at the Virginia Commonwealth University School of Medicine, who is a board-certified clinical toxicologist and has completed the American College of Emergency Physicians Research Academy. Prior instructional experience also shaped the workshop design, including the previously developed and implemented tetanus simulation using a similar pretest-lecture-simulation-posttest structure.

### Curricular Context and Learner Preparation

The Virginia Commonwealth University Health Institutional Review Board approved the study (decision number HM20033165). The curricular context was the third week of a 4-week clinical toxicology elective. Target learners were fourth-year medical students rotating through the elective, though the broader intended audience is medical students during their clinical years of training. A faculty member is required to facilitate the simulation, ideally with training in emergency medicine, internal medicine, or neurology since these specialties most closely align with the clinical content of the case. Facilitators should receive training on operating the simulation manikin and software to ensure they can accurately reflect changes in the patient's condition throughout the session. Required preparation for students to successfully complete the case includes foundational skills in basic airway management and peripheral IV placement, an understanding of the essential elements of a history of present illness for a patient with an overdose, and prior completion of the lecture on carbamazepine toxicity.

### Equipment/Environment

We delivered the lecture in a classroom setting. The required equipment for the classroom included tables, chairs, a computer, and a projection screen. We arranged the room with the students facing the screen and the facilitator facing the students.

We conducted the experiential learning component in a simulation lab room organized into stations: personal prophylaxis, whiteboard, pharmaceutical, simulation manikin, code cart, IV access, control panel, projector, and debrief. The environment was designed to replicate an emergency department. Although the code cart and most of the pharmaceuticals were not required to complete the case, we included them to enhance realism. Additional details are provided in Appendix A.

The high-fidelity manikin offered programmable respiratory, cardiac, and abdominal settings, palpable peripheral pulses, an airway capable of simulating edema, adjustable pupils, and seizure activity, though it could not move independently or be physically manipulated by students. We programmed the manikin to exhibit tachycardia with occasional sobbing. The facilitator connected voice controls and interacted with the participants, portraying a confused patient. Participants viewed a cardiac monitor throughout the scenario. Both the cardiac monitor and manikin responses were adjusted in real time to reflect changes in the patient's condition to the students’ interventions.

### Personnel

The simulation required a facilitator. The facilitator portrayed multiple roles—including the patient's voice, a nurse, and a consultant—and delivered the clinical history to participants. The facilitator also adjusted the manikin's settings throughout the exercise in response to student actions and the evolving scenario. We did not use actors or standardized patients.

### Implementation

We sent learners email invitations to participate voluntarily, emphasizing that participation would not affect their grades. Two weeks before the lecture and simulation, students completed a pretest (Appendix B) to assess their baseline knowledge of carbamazepine and a Likert-scale questionnaire (Appendix C) evaluating their perceived ability to manage a patient with carbamazepine overdose, helping to minimize recall bias. We did not provide students with the answers to the pretest.

The day before the simulation, students attended a 30-minute classroom lecture on carbamazepine (Appendix D). The following day, in small groups of 4-6, they participated in a single simulated case involving a 26-year-old patient with an acute-on-chronic immediate-release carbamazepine overdose in the simulation lab. Students were asked to interview the patient, perform a physical exam, initiate initial management steps, request laboratory studies, provide indicated treatments, and determine patient disposition. We did not require students to place admission orders or write an encounter note. Upon entering the lab, students received patient histories, and facilitators remained present to interact with learners directly.

We prepared the room according to the materials listed in Appendix A and configured the software based on the simulated case (Appendix E). We adjusted manikin responses in real time based on student actions and displayed requested laboratory values and diagnostic images on a television screen (Appendix F). We used a critical actions checklist to evaluate performance during each simulation (Appendix G). We allowed 15 minutes for completion of each case.

Immediately after the simulation, we led a 15-minute structured debriefing session to review learners’ clinical decisions and reinforce key concepts related to carbamazepine pharmacokinetics and toxicokinetics (Appendix H). Students then completed a posttest (see Appendix B), identical to the pretest, and a Likert-scale questionnaire (Appendix J). We gave students the answer key (Appendix I) with explanations after the posttest.

### Debriefing

Learners sat in a semicircle with the facilitator facing them, and the debriefing was divided into 7 sections aligned with the case objectives. We began by discussing team performance, asking students to reflect on their actions and engage in critical thinking about what went well and what could be improved (Section I). We then transitioned to general principles of toxicology, including the key elements of a toxicology history and methods to decrease absorption or enhance elimination, reviewing these concepts to reinforce foundational knowledge and highlighting their relevance to the case (Section II). After establishing these general principles, we focused on the pharmacokinetics and pathophysiology of carbamazepine toxicity to reinforce baseline factual knowledge (Section III). We then reviewed the pertinent history, physical examination findings, and laboratory values, emphasizing how these related to carbamazepine's mechanism of action (Section IV). Building on this, we discussed treatment options, appropriate consultations, and patient disposition (Section V). Following the management discussion, we presented several hypothetical scenarios illustrating alternative clinical presentations of carbamazepine toxicity (Section VI). We concluded with key take-home pearls, including carbamazepine's pregnancy category and on which drug screen it may falsely turn positive (Section VII). For a detailed guideline including question prompts, see Appendix H.

### Assessment and Evaluation

The assessment was multifactorial, using multiple-choice tests, a critical actions checklist, and the New World Kirkpatrick model to assess reactions and learning from the workshop. We measured knowledge acquisition (Kirkpatrick level 2) by comparing pretest and posttest scores (Appendix B). The assessment consisted of a 10-item multiple-choice test addressing indications and contraindications, mechanism of action, pharmacokinetics, and metabolism of carbamazepine, as well as the pathophysiology, physical examination findings, abnormal laboratory values, and management of carbamazepine overdose. All items were equally weighted with scores reported as percentages. All authors contributed to item development, and the questions were reviewed by an emergency medicine physician with fellowship training in medical education to ensure clarity and alignment with the learning objectives. Although the instrument was not formally validated, all learners reported accurately understanding the items. Following the simulation and debriefing session, participants completed the same assessment to measure postsimulation knowledge gains.

We utilized a critical actions checklist (see Appendix G) to evaluate student performance during the simulation (Kirkpatrick level 2, acquisition of knowledge). This checklist was designed to capture essential clinical actions expected during the initial evaluation and management of a patient with carbamazepine overdose. Key elements included obtaining a history of present illness, performing a focused physical examination, and completing initial patient care tasks such as checking a fingerstick blood glucose level, obtaining an electrocardiogram (ECG), establishing IV access, ordering a carbamazepine level and pregnancy test, initiating treatment with multidose activated charcoal, consulting appropriate specialty services, and arranging hospital admission.

Evaluators should be familiar with the critical actions checklist prior to using it to assess student performance. We suggest that evaluators review the checklist and case script in advance of the simulation, and institutions may consider a brief structured orientation or facilitator guide to promote consistency in scoring across evaluators.

We evaluated participant reactions to the workshop (Kirkpatrick level 1) using a Likert-scale questionnaire (see Appendix J). Questionnaire 1, administered immediately after the simulation, assessed participants’ perceptions of case difficulty, appropriateness for their level of training, simulation length, and the value of practicing procedural skills.

In addition to the pre- and posttests, we evaluated learning (Kirkpatrick level 2) with a second Likert-scale questionnaire administered before and after the simulation (see Appendix C and questionnaire 2 in Appendix J). This instrument measured learners’ confidence in identifying clinical manifestations, obtaining and interpreting appropriate diagnostic studies, and initiating treatment for a patient presenting with carbamazepine overdose.

We evaluated learning further with a third postsimulation Likert-scale questionnaire that assessed participants’ intended changes in practice and anticipated transfer of skills to other clinical contexts (see questionnaire 3 in Appendix J). Domains included modifying prior approaches to history taking, adjusting previously performed physical examinations, refining the selection of diagnostic studies for patients with suspected overdose, and applying procedural skills learned during the simulation to other clinical scenarios.

These domains aligned with the overall learning objectives, and we selected this format based on prior validation of similar instruments in assessing self-reported knowledge and confidence. Lastly, we provided students with the opportunity to anonymously submit narrative feedback.

## Results

Between June 2025 and March 2026, a total of 66 fourth-year medical students rotated through the 4-week toxicology elective. Of these, 49 participated in the lecture, simulation, and pre- and posttests. Forty-nine students completed the pre- and posttests, 39 students completed the presimulation Likert-scale questionnaire, and 41 completed the postsimulation Likert-scale questionnaire. All participants attended the required lecture. We conducted the simulation 10 times.

The mean pretest score was 67% (range = 20%-100%, *SD* = 19), and the mean posttest score was 98% (range = 80%-100%, *SD* = 5), as shown in Figure 1. A Wilcoxon signed-rank test demonstrated a statistically significant improvement in scores (W = 0.0, *P* < .001), with a large effect size (*r* = 0.87).

**Figure 1.**
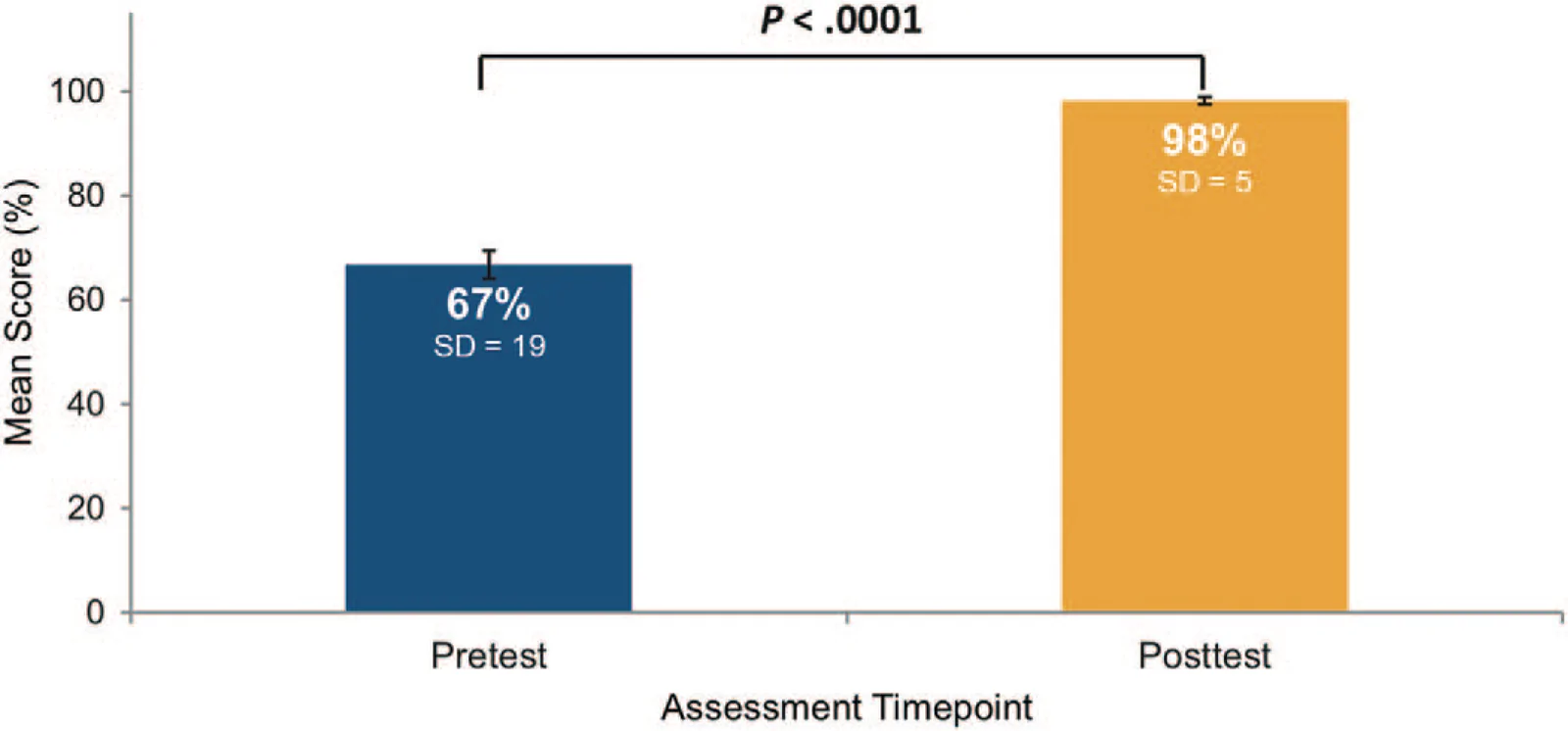
Mean pretest and posttest knowledge scores on a carbamazepine overdose simulation (*N* = 49). Error bars represent *SD* = 1.

All groups completed all critical actions within 15 minutes. Although some teams required occasional prompts or guidance, as outlined in Appendix F, all students ultimately completed every required checklist component. Specifically, 4 groups did not complete a thorough assessment of the patient's coordination and gait, and 3 groups did not administer multidose activated charcoal without prompting.

Median presimulation confidence scores on the Likert-scale questionnaire for identifying clinical manifestations, obtaining and interpreting appropriate diagnostic studies, and initiating treatment were 3, 4, and 3, respectively; all increased to 5 following the simulation. Because surveys were anonymous and not paired by student, we applied a Mann-Whitney U to analyze pre- and postsimulation data. All 4 domains demonstrated statistically significant improvement (*P* < .001 for all). These results are summarized in Figure 2.

**Figure 2.**
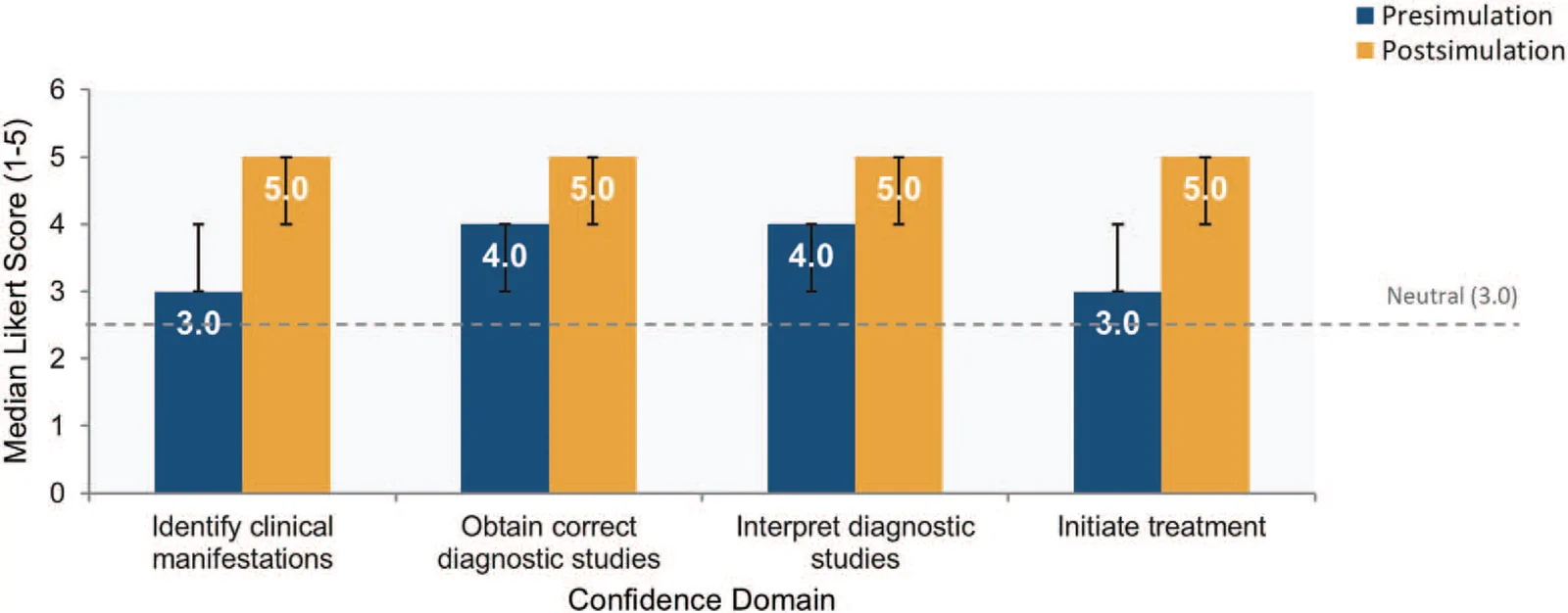
Median self-reported confidence ratings before (*N* = 39) and after (*N* = 41) a carbamazepine overdose simulation with error bars representing interquartile range.

On the Likert-scale questionnaire assessing reactions to the workshop, students rated the simulation experience favorably. All respondents agreed or strongly agreed that the simulation was appropriate for their level of training, that the workshop length was appropriate, and that they learned skills applicable to other areas of medicine. Additionally, 93% valued the opportunity to perform procedural skills, and 90.2% reported feeling appropriately challenged by the case. On the Likert-scale questionnaire assessing intent to change practice, students expressed a strong intention to apply the toxicologic frameworks in future clinical settings. Specifically, 92.7% agreed or strongly agreed that they would use toxicologic history-taking guidelines, and 100% agreed or strongly agreed that they would apply toxicologic physical examination and laboratory/imaging guidelines, as well as the skills learned in the workshop, to other areas of medicine. These results are summarized in Figure 3.

**Figure 3.**
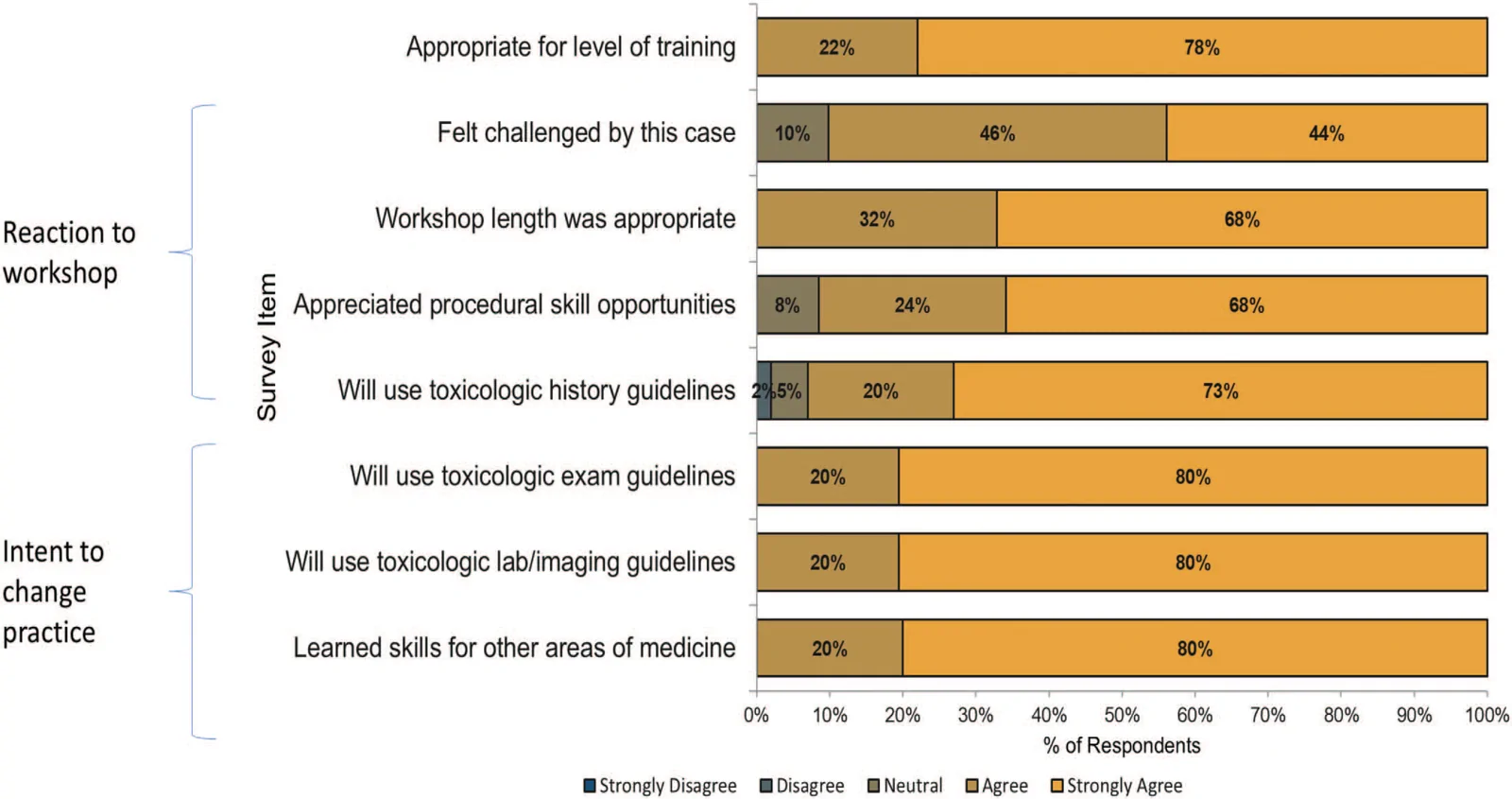
Postsimulation (*N* = 41) responses on reactions to the workshop and intent to change practice.

In their narrative feedback, learners reported that the lecture provided adequate delivery of core content and improved their understanding of how to care for a patient with carbamazepine toxicity. They noted that taking a history of present illness from the simulated patient reinforced general toxicology concepts. Learners also appreciated the opportunity to practice procedural skills, particularly placing a peripheral IV. Lastly, they felt IV placement, airway assessment, toxicologic history taking, ECG interpretation, and triage skills were transferable competencies.

## Discussion

We designed this multimodal teaching session, including a lecture, simulation, and debrief, to present students with the opportunity to manage a real-life example of carbamazepine overdose in a safe learning environment. We aimed to deepen their medical knowledge regarding the drug's pharmacokinetics and available treatment options, and fine-tune their approach to the poisoned patient with specific learning objectives. Achievement of learning objective 1—identifying pharmacokinetics of carbamazepine—was supported by statistically significant improvement in pre- and posttest scores. Achievement of learning objectives 2, 3, and 4 was demonstrated by each group's ability to complete all critical actions within the allotted time frame, including obtaining a focused toxicologic history, performing a targeted physical exam, interpreting clinical and laboratory findings, and implementing evidence-based management. Taken together, the statistically significant improvement in test scores, perceived ability to manage these patients, intent to change practice, and positive reaction to the simulation, we believe that we successfully accomplished this goal.

On reflection, we realized that the simulated patient arrived at the emergency department 16 hours after ingesting carbamazepine, rendering single-dose activated charcoal ineffective as a method of gastrointestinal decontamination. In real-life scenarios, however, patients presenting within the first hour could benefit from this intervention. To address this, we included hypothetical situations during the debrief where single-dose activated charcoal would have been appropriate. The patient was not in critical condition, despite the potential severity of carbamazepine overdose. During the debrief, we discussed seizure risk in critically ill patients and emphasized the use of benzodiazepines for management. We also discussed the potential for hypotension to occur, requiring vasopressor support. This case involved an acute-on-chronic carbamazepine overdose, which does not represent all clinical presentations of carbamazepine toxicity. Accordingly, our debrief discussion addressed how variability in clinical manifestations can arise based on the existing body burden of carbamazepine, differences in CYP3A4 induction status, and concomitant use of medications metabolized by CYP3A4. Throughout the simulation, we noticed that while participants did establish a history of carbamazepine ingestion, their focused histories for poisoned patients were sometimes incomplete. A prebrief could enhance history taking by highlighting key questions—such as what was ingested, the dose, co-ingestions, timing, and intent.

We recognize several limitations to the project. It requires access to a high-fidelity manikin simulation center, adequate space, and adequate funding. It also requires a trained facilitator to operate the high-fidelity manikin to create a realistic simulation. Institutions that may not have access to the same learning materials can still utilize this study with some adjustments. If no high-fidelity manikin is available, we suggest using a trained standardized patient to provide a history and simulate key aspects of the neurological findings. If a phlebotomy manikin is unavailable, students may state that they would like to obtain IV access instead of performing this procedure. Given the nature of the case itself, the generalizability of the case is best applied to specialties such as emergency medicine, internal medicine, toxicology, and neurology, though it does not exclude generalizability to other specialties. Additionally, this study may not be generalizable to other institutions depending on the different curricula offered at each school. This study also lacks generalizability to medical students in different stages of training, as fourth year medical students tend to have additional clinical experience. We acknowledge that the results of this study are subject to selection bias due to the participation of students who have chosen to enroll in the course and have volunteered to participate in this study. Students enrolled in the clinical toxicology elective had interests in nonsurgical specialties such as emergency medicine, internal medicine, and pediatrics. Currently, the principal investigator is the sole user of this simulation. Although we did not conduct formal evaluator training, evaluators should be familiar with the critical actions checklist prior to implementing the simulation. Because participation was voluntary and without impact on grades, we have not developed a formal remediation process.

We also recognize the possibility of test-retest effects and recall bias influencing posttest outcomes. While an attempt was made to mitigate this effect by withholding test answers and a 2-week time gap between the pre and posttest, there is a possibility that the high posttest average can be partially attributed to recall bias. Future iterations of this study can consider prolonging the interval between pretest and posttest to mitigate this bias. While the pretest and posttest were paired, we did not pair the Likert-scale results; in hindsight, using a unique identifier would have allowed us to align individual pre- and postsimulation responses. The Likert-scale questionnaire had a lower response rate on the pretest and posttest scores, likely because we used a different assessment method. Students completed the pretests and posttests in Canvas, which they already used for the toxicology course, whereas we administered the Likert-scale questionnaire through Google Forms. The Likert-scale questionnaire relied on self-reporting rather than objective performance measures, which may have caused response bias. Lastly, we assessed short-term knowledge gain only.

We plan to continue this simulation as part of our clinical toxicology elective. Our institution also offers a 2-week online toxicology elective, and we incorporated the carbamazepine lecture and associated quiz into that curriculum. We are currently running the project in a similar pretest-posttest format, with the lecture serving as the sole intervention between assessments. We plan to expand the project by comparing test gains from the simulation and lecture cohort with the lecture-only cohort in both short- and long-term assessments.

We plan to expand the multimodal learning with simulation into both emergency medicine and neurology training at VCU Health. Looking ahead, we plan to include residents and vary the patient's presentation—particularly consciousness and hemodynamic stability following ingestion—to give learners opportunities to practice toxicology history taking, administer activated charcoal, utilize hemodialysis, place central and arterial lines, and manage airway interventions such as intubation.

## Appendices


Stations and Equipment.docxPretest-Posttest.docxPresimulation Likert Questionnaire.docxCarbamazepine Lecture.pptxSimulated Patient Case.docxLab Values and Imaging.docxCritical Actions Checklist.docxCarbamazepine Debrief.pptxAnswer Key.docxPostsimulation Likert Questionnaire.docx
*All appendices are peer reviewed as integral parts of the Original Publication.*
