# Supplementary material for: Acute-on-Chronic Carbamazepine Overdose: A Toxicology Simulation Case for Fourth-Year Medical Students
Source: MedEdPORTAL. 2026 Oct 14;22:11640. doi: 10.15766/mep_2374-8265.11640 (PMC13685907; doi:10.15766/mep_2374-8265.11640)
Supplement: Supplementary file 1 [file mep_2374-8265.11640-s001.zip › A. Stations and Equipment.docx]

Appendix A: Stations and Equipment

Pictures are owned by author Michelle Troendle.

**Station 1 - The personal prophylaxis station**

- Sink
- Soap
- Hand sanitizer
- Disposable paper towels
- Fire blanket
- Gloves (XS, S, M, L, XL)
- Goggles
- Face shields
- Sharps dispenser
- Sterile gowns and gloves
- Foot coverings

**Station 2 - The whiteboard station**

- Whiteboard
- Dry erase markers
- Dry erase cleaner with eraser

**Station 3 - The pharmaceutical station**

- Table

- Various pharmacological medications
  - Intravenous fluid (IVF)
  - Vasopressors
  - Antidotes
  - Benzodiazepines
  - Rescue medication (Intralipid)
  - Electrolyte repletion

**Station 4 - The simulation manikin**

- Manikin
- Sheet
- Monitor
- Sphygmomanometer
- Wall suction with Yankauer and collection kit
- Oxygen with tapered oxygen connector
- IV pole with pump
- Pulse oximeter

**Station 5 - The code cart**

- Airway cart
  - Nasal cannulas
  - Capnography
  - Non-rebreather masks
  - Variously sized uncuffed/cuffed endotracheal tubes
  - Variously sized Mac and Miller blades
  - Glidescope with rigid stylet
  - Ambu bag
  - Bougie
- Pharmacy
  - Epinephrine
  - Adenosine
  - Atropine
  - Sodium bicarbonate
  - Dextrose 10%
- Defibrillator/pacer with pads and monitor
- Backboard

**Station 6 - The IV access station**

- IV arm

- Pressurized venipuncture arm mock blood supply
- 10 cc flush
- IV start kit
  - Tegaderm
  - Chlorhexidine
  - Tourniquet
  - Identifying info sticker
  - 2x2 gauze-sponge
- Supplemental alcohol swab
- Tape
- 22-gauge IVs

**Station 7 - The control panel station**

- Computer that adjusted manikin and cardiac monitor functions
- Table
- Chair

**Station 8 - The projector station**

- Wall screen to display results

**Station 9 - The debrief station**

- 6 chairs (5 for students and 1 for instructor)

- Table
