# Supplementary material for: Acute-on-Chronic Carbamazepine Overdose: A Toxicology Simulation Case for Fourth-Year Medical Students
Source: MedEdPORTAL. 2026 Oct 14;22:11640. doi: 10.15766/mep_2374-8265.11640 (PMC13685907; doi:10.15766/mep_2374-8265.11640)
Supplement: Supplementary file 1 [file mep_2374-8265.11640-s001.zip › B. Pretest-Posttest.docx]

**Appendix B: Pretest/Posttest**

1. Which symptom category is most commonly associated with a carbamazepine overdose?
   a. Renal symptoms (e.g., increased urine output)
   b. Pulmonary symptoms (e.g., shortness of breath, cough)
   c. Central nervous system symptoms (e.g., drowsiness, ataxia, seizures)
   d. Dermatologic symptoms (e.g., rash, bullous lesions)
2. Which of the following symptoms would most likely appear when carbamazepine serum levels are between 12–15 mg/L (normal therapeutic serum 4-12 mg/L)?
   a. Disorientation and ataxia
   b. Combativeness and hallucinations
   c. Seizures and coma
   d. Cardiac arrest
3. All of the following are indications for carbamazepine **except**:
   a. Seizure disorder
   b. First-line use in pregnancy for seizure disorder
   c. Trigeminal neuralgia
   d. Bipolar disorder
4. Which of the following most accurately describes a mechanism by which carbamazepine exerts its anticonvulsant effects?
   a. Agonism of N-methyl-D-aspartate (NMDA) receptors
   b. Inhibition of Gamma-Aminobutyric Acid (GABA) transaminase, reducing GABA catabolism
   c. Inhibition of voltage-gated N-type calcium channels, decreasing glutamate release
   d. Inhibition of voltage-gated sodium channels, decreasing glutamate release
5. Which of the following statements regarding carbamazepine is correct?
   a. Carbamazepine has rapid and predictable absorption
   b. Carbamazepine has a broad therapeutic window
   c. Carbamazepine’s metabolism varies widely due to genetic differences and interactions with other xenobiotics
   d. Carbamazepine metabolism is not influenced by cytochrome P450 enzymes
6. Which of the following best describes the primary metabolic pathway for carbamazepine?
   a. Renal filtration followed by conjugation
   b. Direct glucuronidation in the intestine
   c. Hydrolysis by plasma esterases to an inactive form
   d. Oxidation by cytochrome P450 3A4 (CYP3A4)
7. Which metabolite of carbamazepine is pharmacologically active?
   a. 2-hydroxycarbamazepine
   b. 3-hydroxycarbamazepine
   c. Carbamazepine glucuronide
   d. Carbamazepine-10,11-epoxide
8. What is the mechanism by which multiple‑dose activated charcoal enhances the elimination of carbamazepine during an overdose?
   a. Enhances renal clearance of carbamazepine
   b. Binds carbamazepine in the gastrointestinal tract, reducing reabsorption and facilitating enterohepatic clearance
   c. Neutralizes carbamazepine in the bloodstream
   d. Inhibits cytochrome P450 metabolism of carbamazepine
9. Which intervention for enhancing the elimination of carbamazepine should be performed in cases of intractable seizures or life‑threatening dysrhythmias?
   a. Single dose of activated charcoal
   b. Gastric lavage
   c. Urinary alkalinization
   d. Hemodialysis
10. Which of the following drug screens can carbamazepine turn positive?
    a. Tricyclic antidepressant
    b. Opioid
    c. Cocaine
    d. Benzodiazepine
