# Supplementary material for: Acute-on-Chronic Carbamazepine Overdose: A Toxicology Simulation Case for Fourth-Year Medical Students
Source: MedEdPORTAL. 2026 Oct 14;22:11640. doi: 10.15766/mep_2374-8265.11640 (PMC13685907; doi:10.15766/mep_2374-8265.11640)
Supplement: Supplementary file 1 [file mep_2374-8265.11640-s001.zip › C. Presimulation Likert Questionnaire.docx]

**Appendix C: Presimulation Likert-Scale Questionnaire**

**Presimulation Questionnaire - Assessment of Learning (Kirkpatrick Level 2)**

Rate your agreement with the following statements using a five-point Likert scale (Strongly Disagree, Disagree, Neutral, Agree, Strongly Agree):

|  | **Strongly Disagree** | **Disagree** | **Neutral** | **Agree** | **Strongly Agree** |
| --- | --- | --- | --- | --- | --- |
| I can accurately recognize the clinical manifestations associated with carbamazepine overdose. |  |  |  |  |  |
| I can obtain the appropriate diagnostic studies for a patient presenting with carbamazepine overdose. |  |  |  |  |  |
| I can accurately interpret the diagnostic studies obtained for a patient with carbamazepine overdose. |  |  |  |  |  |
| I can initiate appropriate treatment for a patient with carbamazepine overdose. |  |  |  |  |  |
