# Supplementary material for: Acute-on-Chronic Carbamazepine Overdose: A Toxicology Simulation Case for Fourth-Year Medical Students
Source: MedEdPORTAL. 2026 Oct 14;22:11640. doi: 10.15766/mep_2374-8265.11640 (PMC13685907; doi:10.15766/mep_2374-8265.11640)
Supplement: Supplementary file 1 [file mep_2374-8265.11640-s001.zip › E. Simulated Patient Case.docx]

| **Appendix E: *MedEdPORTAL* Simulation Case Template**  **SIMULATION CASE TITLE: Carbamazepine Overdose**  **AUTHORS:** Michelle Troendle, MD, Caelan Nzamba, BS, Lauren Leung, BS, Johnny Bui, BS, John Kothmann, MCS, Derick Nguyen, BS, Krithika Senthil, BSPH  **LEARNER AUDIENCE:** Fourth-Year Medical Students on Critical Care Toxicology Elective | |
| --- | --- |
| **PATIENT NAME:** Colleen Jones  **PATIENT AGE:** 26-year-old  **CHIEF COMPLAINT:** “I’m having trouble walking and I’m having palpitations after taking too much carbamazepine”  **PHYSICAL SETTING:** Adult Emergency Department | |
| Instructions: This template serves as a guide on how to create and run the case. Learners have 15 minutes to complete all critical actions and admit the patient. | |
| **Brief Narrative Description of Case** | This case involves a 26 year-old female (she/her/hers) with a history of migraines and seizure disorder who presents to the emergency department via EMS with palpitations and difficulty walking after intentionally ingesting 20 tablets of carbamazepine in the setting of self-harm. She is drowsy and tearful with a Glasgow Coma Scale (GCS) of 14. Physical examination reveals tachycardia, ataxia, horizontal nystagmus, mild tremor, and multiple lacerations on the left forearm. The goals of this case are to identify and manage a simulated patient with carbamazepine toxicity. |
| **Primary Learning Objectives** | - Obtain a history of present illness related to toxic ingestion - Recognize physical exam findings of carbamazepine toxicity - Order and interpret diagnostic labs including carbamazepine level - Administer multidose activated charcoal - Coordinate care with admitting services and consulting services |
| **Critical Actions** | - Obtain history of carbamazepine overdose - GCS assessment - Coordination assessment - Finger stick blood glucose (FSBG) - Electrocardiogram (ECG) - Establish intravenous (IV) access - Start IV fluids - Order carbamazepine level - Order pregnancy test - Administer multi-dose activated charcoal (MDAC) - Consult toxicology - Consult psychiatry - Admit to internal medicine or neurology |
| **Learner Preparation or Prework** | Learning preparation included training in basic airway management, instruction on establishing IV access, attending a lecture on carbamazepine, and reviewing the key components needed to obtain a thorough toxicology history and physical exam. |

| **Initial Presentation** | | | |
| --- | --- | --- | --- |
| **Initial Vital Signs** | Oral Temp 36.1 °C (96.9 °F), **HR 123 bpm**, RR 12 br/min, SpO2 100 %, BP 116/78 mmHg | | |
| **Overall Setting and Appearance** | The simulation setting is an adult emergency department examination room. The patient is brought in by EMS and is lying on a hospital stretcher. The patient is drowsy and tearful. | | |
| **Standardized Participants (and Their Roles in the Room at Case Start)** | The patient (manikin) is lying on the hospital stretcher. The patient’s voice is controlled by the facilitator. The patient answers questions regarding the history of present illness, extended history, and review of systems. The facilitator sits at a control panel and alters the patient/manikin settings. The facilitator also plays the role of the bedside nurse. If an exam finding was not demonstrable by the manikin (i.e. gait), the facilitator provided the results upon request. | | |
| **HPI** | The following information should be volunteered by the facilitator:  This is a 26-year old patient presenting to the emergency department with the complaint of overdose.  The following information should be obtained by the learners:  The patient is a 26-year-old female with a history of migraines and seizure disorder who presents to the emergency department with palpitations and difficulty walking, 16 hours after intentionally ingesting approximately twenty 100 mg tablets of carbamazepine in a self-harm attempt. She denies any co-ingestions. Her symptoms include drowsiness, difficulty walking, and palpitations. She denies fevers, chills, nausea, vomiting, headache, rash, chest pain, or shortness of breath. Her last menstrual cycle was 2 weeks ago, and she uses an intrauterine device (IUD) for contraception. | | |
| **Past Medical/Surgical History** | **Medications** | **Allergies** | **Family History** |
| Migraines, seizure disorder  **Social Hx:** Is sexually active with 1 male partner; does not smoke, drink alcohol, or use recreational drugs | Carbamazepine Immediate Release 100 mg QID | None | Mother - migraines  Father - hypertension |
| **Physical Examination** | | | |
| **General** | **Drowsy, tearful and in mild distress; awake, mildly tremulous,** normal body habitus | | |
| **HEENT** | Normocephalic, atraumatic, **+ horizontal nystagmus,** pupils 4 mm and reactive, extraocular movements and visual fields intact, moist mucous membranes | | |
| **Neck** | Supple, no bruits, trachea midline, no cervical spine step-offs or tenderness to palpation | | |
| **Lungs** | Non-labored breathing; equal breath sounds bilaterally; clear to auscultation in all lung fields | | |
| **Cardiovascular** | **Tachycardic** with regular rhythm; 2+ carotid, femoral, radial, brachial, dorsalis pedis, and posterior tibial pulses bilaterally; no murmurs, rubs, or gallops | | |
| **Chest Wall** | No deformities | | |
| **Breast** | No masses, skin changes, or dimpling | | |
| **Back** | Normal alignment; no step-offs or tenderness to palpation | | |
| **Musculoskeletal** | No edema or deformities | | |
| **Gastrointestinal** | Normoactive bowel sounds, soft, non-distended, non-tender, no rebound or guarding | | |
| **Neurological** | **GCS 14 (E3V5M6)**  **Drowsy** but awake, oriented to person, place, time, and situation  Gait**: + ataxia**  Coordination: **+ past-pointing with finger-to-nose, heel-to-shin are not smooth in nature**  **Cranial Nerves: + horizontal nystagmus** (otherwise CN II-XII grossly intact)  Reflexes: 2+ brachioradialis, bicep, tricep, patellar, and ankle reflexes bilaterally  Sensation: Light touch sensation intact to upper and lower extremities bilaterally  Motor strength: 5/5 motor strength to upper and lower extremities bilaterally | | |
| **Skin** | Warm, dry, intact without rashes, ecchymosis or pallor | | |
| **GU** | Normal female external genitalia | | |
| **Psychiatric** | **Mildly slow to respond, + impulsive, depressed mood, withdrawn with blunt affect, + suicidal ideations** | | |

| **Instructor Notes - Changes and CASE Branch Points** | | |
| --- | --- | --- |
| **Intervention / Time Point** | **Change in Case** | **Additional Information** |
| Participants request FSBG within 3 minutes of case | If participants request FSBG, it returns as 78 mg/dL. | If participants do not obtain FSBG, RN prompts, “The patient is drowsy. Are there any bedside tests we can order to rule out reversible causes of drowsiness?” |
| Participants request ECG within 3 minutes of case | If ECG is obtained there is no change in vital signs. | If participants do not order ECG, RN prompts, “The heart rate is fast. Is there any bedside test that can assess the heart rhythm?” |
| Participants request neurologic exam within 8 minutes of case | If neurological exam is requested, participants find a GCS 14, ataxia, mild tremor, horizontal nystagmus, positive past-pointing with finger-to-nose test, and movements of heel to shin are not smooth in nature | If participants do not perform a neurologic exam, the facilitator prompts, “What abnormalities on physical exam are seen in carbamazepine overdose?” |
| Participants administer IV fluids for tachycardia within 10 minutes of case | If IV fluids are administered, the patient’s heart rate improves to 110 bpm.  If IV fluids are not administered, there is no improvement in tachycardia. | If participants do not administer IV fluids, the facilitator prompts, “The patient has tachycardia, is there anything you could give to alleviate this?” |
| Participants request carbamazepine level within 12 minutes of case | If participants request a carbamazepine level, it returns as 14.9 mg/L. | If a carbamazepine level is not ordered, RN states, “The patient took a lot of carbamazepine. Is there a test you could order to assess her level of intoxication?” |
| Participants must administer MDAC within 13 minutes of case | If participants administer MDAC, there is no change in stabilization of vital signs.  If participants do not administer MDAC, the patient has an increase in tachycardia to 135 bpm and worsening ataxia and nystagmus. | If participants do not administer MDAC, the facilitator asks, “The patient appears to be worsening. Is there a way to enhance the elimination of this toxin?” |
| Participants call the internal medicine or neurology team for admission with a toxicology consult, psychiatry consult, and 1:1 sitter within 15 minutes of the case | If the participants request internal medicine or neurology team admission, the patient is accepted to this service.  If participants call for admission to any other service, the patient is declined for admission. | If participants do not recognize that the patient needs a psychiatry consult, toxicology consult, and 1:1 sitter, the facilitator asks, “What consulting services can be considered? Should this patient have suicide precautions?” |

**Ideal Scenario Flow**

Before entering the simulation room, the learners delegate the following roles: case leader, IV placement, scribe, airway management, and pharmacy. Upon entering, participants observe a tearful 26-year-old female on a stretcher who reports an intentional overdose of carbamazepine.

The team places the patient in the sniffing position and on the cardiac monitor and obtains IV access, a FSBG, and an ECG. They then perform a focused toxicologic history and physical exam emphasizing cardiac, respiratory, and neurological systems. The ECG results become available and they interpret it as sinus tachycardia without ST changes. They administer IV fluids for sinus tachycardia.

Recognizing the patient’s intentional ingestion of carbamazepine, self-harm is confirmed and the team requests a 1:1 sitter and psychiatry consult as well as a toxicology consult. If learners do not assess suicidal ideation or request suicide precautions, the psychiatry consultant would inquire whether suicidal ideation has been assessed and recommend one-to-one observation (sitter) before accepting the patient for admission.

The team orders labs including CBC, BMP, liver function tests, ethanol level, acetaminophen level, salicylate level, carbamazepine level, beta-HCG pregnancy test, and urinalysis. Labs are significant for leukopenia, hyponatremia, and an elevated carbamazepine level of 14.9 mg/L. Urinalysis is significant for dark color and high specific gravity. They elect to administer multiple doses of activated charcoal orally to enhance elimination of carbamazepine while closely monitoring her airway and explain to the patient the importance of this clinical intervention to enhance elimination. They counsel the patient regarding her laboratory abnormalities and that they are expected to normalize as the toxicity abates.

Lastly, participants call internal medicine or neurology, and the patient is accepted for admission.

**Anticipated Management Mistakes**

1. Incomplete Neurological Assessment - Omission of Gait Evaluation: Students often requested the patient’s motor strength, sensory function, and cranial nerves, but neglected to evaluate gait. The facilitator prompted discussion on the importance of gait assessment for evaluating cerebellar function and ataxia in the setting of carbamazepine toxicity.
2. Underrecognition of hyponatremia: This case featured mild hyponatremia, which was overlooked by several students. The facilitator guided a discussion on the potential for carbamazepine to induce syndrome of inappropriate antidiuretic hormone secretion (SIADH) and by increasing antidiuretic hormone receptors in the kidney.
3. Failure to administer MDAC for enhanced elimination: In this case, carbamazepine levels were mildly elevated and several students did not consider administration of multiple doses of activated charcoal for enhanced elimination. The facilitator emphasized the patient’s symptomatic presentation and carbamazepine’s narrow therapeutic index, making MDAC an appropriate treatment option despite mildly elevated carbamazepine levels.
4. Failure to order urinalysis: Urinalysis was occasionally omitted from the initial workup. The facilitator used this oversight to underscore the role of urinalysis in evaluating suspected SIADH as it can reveal the characteristic findings of concentrated urine with high specific gravity and dark yellow color.
