# Supplementary material for: Acute-on-Chronic Carbamazepine Overdose: A Toxicology Simulation Case for Fourth-Year Medical Students
Source: MedEdPORTAL. 2026 Oct 14;22:11640. doi: 10.15766/mep_2374-8265.11640 (PMC13685907; doi:10.15766/mep_2374-8265.11640)
Supplement: Supplementary file 1 [file mep_2374-8265.11640-s001.zip › F. Lab Values and Imaging.docx]

**Appendix F: Laboratory Values and Imaging**

**Complete blood count**

Result	Reference

WBC	**4.3**	4.5-11 x10e9/L

Hgb	13.6	12-16 g/dL

Hct	38.9	36-46%

Plt	225	150-450 x10e9/L

**Basic metabolic panel**

Result	Reference

Sodium	**132**	136-145 mmol/L

Potassium	4.4	3.5-5.1 mmol/L

Chloride	101	98-107 mmol/L

CO_2_	23	22-30 mmol/L

BUN	15	6-20 mg/dL

Creatinine	0.7	0.6-1.2 mg/dL

Glucose	78	65-99 mg/dL

Calcium	8.9	8.0-10.6 mg/dL

**Hepatic panel**

Result	Reference

Albumin	3.9	3.5-5.0 g/dL

T bili	0.7	0.0-1.0 mg/dL

D bili	0.1	0.0-0.2 mg/dL

Alk phos	49	35-109 U/L

AST	34	0-37 U/L

ALT	28	0-41 U/L

Total protein	6.9	6.4-8.3 g/dL

**Coagulation profile**

Result	Reference

INR	1.0	< 1.1

PTT	22.8	22-33 sec

**Urinalysis**

Result	Reference

Color	**Dark yellow**	Yellow

pH	5.9	4.5-8

Specific gravity	**1.040**	1.005-1.025

Glucose	None	≤20 mg/dL

Ketones	None	< 3 mg/dL

Nitrites	Negative	Negative

Leukocyte esterase	Negative	Negative

Bilirubin	Negative	Negative

Urobilinogen	0.5	0.5-1 mg/dL

Blood	Negative	Negative

Protein	Negative	≤30 mg/dL

RBCs	0	≤2 RBCs/hpf

WBCs	0	≤2-5 WBCs/hpf

Bacteria	Negative	None

**Urine pregnancy**: Negative

**Carbamazepine: 14.9 mg/L** (normal range 4-12 mg/L)

**EKG** – 124 bpm, QRS 80, QTc 439

**Ewingdo. *ECG Sinus Tachycardia 125 bpm*. Wikimedia Commons. Published November 21, 2020. Accessed May 1, 2025. https://commons.wikimedia.org/wiki/File:ECG_Sinus_Tachycardia_125_bpm.jpg**

**CT Head (if ordered)**: No acute process
