# Supplementary material for: Acute-on-Chronic Carbamazepine Overdose: A Toxicology Simulation Case for Fourth-Year Medical Students
Source: MedEdPORTAL. 2026 Oct 14;22:11640. doi: 10.15766/mep_2374-8265.11640 (PMC13685907; doi:10.15766/mep_2374-8265.11640)
Supplement: Supplementary file 1 [file mep_2374-8265.11640-s001.zip › G. Critical Actions Checklist.docx]

**Appendix G: Critical Actions Checklist**

| **Critical Action** | **Performed** | **Not Performed** |
| --- | --- | --- |
| Obtain history of carbamazepine overdose |  |  |
| Request Glasgow coma score assessment |  |  |
| Request coordination assessment |  |  |
| Order finger stick blood glucose |  |  |
| Order electrocardiogram |  |  |
| Establish intravenous (IV) access |  |  |
| Administer IV fluids |  |  |
| Order carbamazepine level |  |  |
| Order pregnancy test |  |  |
| Administer multidose activated charcoal |  |  |
| Consult toxicology |  |  |
| Consult psychiatry |  |  |
| Admit to internal medicine or neurology |  |  |
