# Supplementary material for: Acute-on-Chronic Carbamazepine Overdose: A Toxicology Simulation Case for Fourth-Year Medical Students
Source: MedEdPORTAL. 2026 Oct 14;22:11640. doi: 10.15766/mep_2374-8265.11640 (PMC13685907; doi:10.15766/mep_2374-8265.11640)
Supplement: Supplementary file 1 [file mep_2374-8265.11640-s001.zip › I. Answer Key.docx]

**Appendix I: Answer Key**

1. Which symptom category is most commonly associated with a carbamazepine overdose?
   a. Renal symptoms (e.g., increased urine output)
   b. Pulmonary symptoms (e.g., shortness of breath, cough)
   c. **Central nervous system symptoms (e.g., drowsiness, ataxia, seizures)**
   d. Dermatologic symptoms (e.g., rash, bullous lesions)

The answer is **c**. Acute carbamazepine toxicity primarily affects the nervous system and cardiovascular system. Neurologic effects include nystagmus, ataxia, seizures, and coma, with some cases progressing to status epilepticus. Cardiovascular effects may involve sinus tachycardia, hypotension, myocardial depression, and, in rare cases, conduction abnormalities such as prolonged QRS complex and QT interval. Carbamazepine may cause syndrome of inappropriate antidiuretic hormone secretion (SIADH); however, this results in water retention and therefore *reduced* urine output.

2. Which of the following symptoms would most likely appear when carbamazepine serum levels are between 12–15 mg/L (normal therapeutic serum 4-12 mg/L)?
   a. **Disorientation and ataxia**
   b. Combativeness and hallucinations
   c. Seizures and coma
   d. Cardiac arrest

The answer is **a**. When carbamazepine serum levels are between 12–15 mg/L, which is slightly above the upper limit of the therapeutic range (4–12 mg/L), mild to moderate toxicity symptoms may begin to appear. The most likely symptoms at this level include:

- Drowsiness
- Dizziness or ataxia (loss of coordination)
- Slurred speech
- Nystagmus (involuntary eye movements)

These symptoms are generally neurologic in nature and reflect early signs of toxicity. Severe effects like seizures, coma, or cardiovascular complications are more commonly associated with significantly higher serum levels (typically >25 mg/L).

3. All of the following are indications for carbamazepine **except**:
   a. Seizure disorder
   b. **First-line use in pregnancy for seizure disorder**
   c. Trigeminal neuralgia
   d. Bipolar disorder

The answer is **b**. Carbamazepine is commonly prescribed for seizure disorders, trigeminal neuralgia, and bipolar disorder. However, it is **not** a first-line treatment for seizure disorders during pregnancy due to its potential teratogenic effects,including neural tube defects and craniofacial abnormalities. Due to this, it is a category D pregnancy drug. In pregnant individuals, alternative medications are typically prescribed to manage seizures.

4. Which of the following most accurately describes a mechanism by which carbamazepine exerts its anticonvulsant effects?
   a. Agonism of N-methyl-D-aspartate (NMDA) receptors
   b. Inhibition of Gamma-Aminobutyric Acid (GABA) transaminase, reducing GABA catabolism
   c. Inhibition of voltage-gated N-type calcium channels, decreasing glutamate release
   d. **Inhibition of voltage-gated sodium channels, decreasing glutamate release**

The answer is **d**. Carbamazepine inhibits sodium (Na⁺) channels in presynaptic neurons, preventing the release of glutamate, an excitatory neurotransmitter. It also inhibits *L-type* calcium channels, decreasing glutamate release.

5. Which of the following statements regarding carbamazepine is correct?
   a. Carbamazepine has rapid and predictable absorption
   b. Carbamazepine has a broad therapeutic window
   c. **Carbamazepine’s metabolism varies widely due to genetic differences and interactions with other xenobiotics**
   d. Carbamazepine metabolism is not influenced by cytochrome P450 enzymes

The answer is **c**. Carbamazepine has slow and unpredictable absorption after oral administration. It has a narrow therapeutic index. It is primarily metabolized by cytochrome P450 3A4 (CYP3A4), and predisposes to drug-drug interactions. Due to the above, therapeutic drug monitoring is required.

6. Which of the following best describes the primary metabolic pathway for carbamazepine?
   a. Renal filtration followed by conjugation
   b. Direct glucuronidation in the intestine
   c. Hydrolysis by plasma esterases to an inactive form
   d. **Oxidation by cytochrome P450 3A4 (CYP3A4)**

The answer is **d**. Carbamazepine itself is not an active xenobiotic. After absorption, it is oxidized by the CYP3A4 into its active form, carbamazepine-10,11-epoxide.

7. Which metabolite of carbamazepine is pharmacologically active?
   a. 2-hydroxycarbamazepine
   b. 3-hydroxycarbamazepine
   c. Carbamazepine glucuronide
   d. **Carbamazepine-10,11-epoxide**

The answer is **d**. The cytochrome P450 enzyme CYP3A4 metabolizes carbamazepine, an inactive parent compound, into its active metabolite, carbamazepine-10,11-epoxide.

8. What is the mechanism by which multiple‑dose activated charcoal enhances the elimination of carbamazepine during an overdose?
   a. Enhances renal clearance of carbamazepine
   b. **Binds carbamazepine in the gastrointestinal tract, reducing reabsorption and facilitating enterohepatic clearance**
   c. Neutralizes carbamazepine in the bloodstream
   d. Inhibits cytochrome P450 metabolism of carbamazepine

The answer is **b**. After carbamazepine is *absorbed* from the gastrointestinal tract, part of the drug is excreted into the bile and then *reabsorbed* in the intestines through enterohepatic recirculation. In an overdose, giving multiple doses of activated charcoal helps by binding carbamazepine within the GI tract, interrupting this recirculation. This prevents the drug from being *reabsorbed* and increases its overall elimination.

9. Which intervention for enhancing the elimination of carbamazepine should be performed in cases of intractable seizures or life‑threatening dysrhythmias?
   a. Single dose of activated charcoal
   b. Gastric lavage
   c. Urinary alkalinization
   d. **Hemodialysis**

The answer is **d**. A single dose of activated charcoal can reduce carbamazepine absorption when administered soon after ingestion, ideally within the first hour. Gastric lavage is another method to limit absorption, although it is now rarely used because of its high complication rate. Urinary alkalinization does not enhance carbamazepine elimination. Because carbamazepine is highly lipophilic and extensively protein‑bound (80–85%), it is not efficiently removed by hemodialysis. However, in cases of severe toxicity, hemodialysis may still be considered as part of the management strategy.

10. Which of the following drug screens can carbamazepine turn positive?
    a. **Tricyclic antidepressant**
    b. Opioid
    c. Cocaine
    d. Benzodiazepine

The answer is **a**. Carbamazepine can turn a tricyclic antidepressant drug screen positive due to its tricyclic chemical structure:

Image created by John Kothman, co-author
