# Supplementary material for: Acute-on-Chronic Carbamazepine Overdose: A Toxicology Simulation Case for Fourth-Year Medical Students
Source: MedEdPORTAL. 2026 Oct 14;22:11640. doi: 10.15766/mep_2374-8265.11640 (PMC13685907; doi:10.15766/mep_2374-8265.11640)
Supplement: Supplementary file 1 [file mep_2374-8265.11640-s001.zip › J. Postsimulation Likert Questionnaire.docx]

**Appendix J: Postsimulation Likert Scale Questionnaire**

**Questionnaire 1 - Assessment of Reactions to the Simulation (Kirkpatrick Level 1)**

Rate your agreement with the following statements using a five-point Likert scale (Strongly Disagree, Disagree, Neutral, Agree, Strongly Agree):

|  | **Strongly Disagree** | **Disagree** | **Neutral** | **Agree** | **Strongly Agree** |
| --- | --- | --- | --- | --- | --- |
| This simulation was appropriate for my level of training. |  |  |  |  |  |
| I felt challenged by this case. |  |  |  |  |  |
| The length of the simulation was appropriate. |  |  |  |  |  |
| I appreciated the opportunity to perform procedural skills. |  |  |  |  |  |

**Questionnaire 2 - Assessment of Learning (Kirkpatrick Level 2)**

Rate your agreement with the following statements using a five-point Likert scale (Strongly Disagree, Disagree, Neutral, Agree, Strongly Agree):

|  | **Strongly Disagree** | **Disagree** | **Neutral** | **Agree** | **Strongly Agree** |
| --- | --- | --- | --- | --- | --- |
| I can accurately recognize the clinical manifestations associated with carbamazepine overdose. |  |  |  |  |  |
| I can obtain the appropriate diagnostic studies for a patient presenting with carbamazepine overdose. |  |  |  |  |  |
| I can accurately interpret the diagnostic studies obtained for a patient with carbamazepine overdose. |  |  |  |  |  |
| I can initiate appropriate treatment for a patient with carbamazepine overdose. |  |  |  |  |  |

**Questionnaire 3 - Assessment of Practice Changes to the Workshop (Kirkpatrick Level 2)**

Rate your agreement with the following statements using a five-point Likert scale (Strongly Disagree, Disagree, Neutral, Agree, Strongly Agree):

|  | **Strongly Disagree** | **Disagree** | **Neutral** | **Agree** | **Strongly Agree** |
| --- | --- | --- | --- | --- | --- |
| I will refine my approach to obtaining a history of present illness in patients who have overdosed and apply it to future overdose cases. |  |  |  |  |  |
| I will refine my approach to performing a physical exam in patients who have overdosed and apply it to future overdose cases. |  |  |  |  |  |
| I will refine the diagnostic studies I order in patients who have overdosed and apply this approach to future overdose cases. |  |  |  |  |  |
| I learned skills throughout these cases that I can apply to other areas of medicine. |  |  |  |  |  |
| If “agree” or “strongly agree”, which areas? | | | | | |
| Please provide any other feedback here: | | | | | |
|  |  |  |  |  |  |
